# Genetic diversity, haplotype analysis, and prevalence of Hepatitis B virus MHR mutations among isolates from Kenyan blood donors

**DOI:** 10.1371/journal.pone.0291378

**Published:** 2023-11-14

**Authors:** Benard Kibet Langat, Kevin Omondi Ochwedo, Jamie Borlang, Carla Osiowy, Alex Mutai, Fredrick Okoth, Edward Muge, Anton Andonov, Elijah Songok Maritim

**Affiliations:** 1 Department of Medical Biochemistry, University of Nairobi, Nairobi, Kenya; 2 Department of Biology, Faculty of Science and Technology, University of Nairobi, Nairobi, Kenya; 3 National Microbiology Laboratory, Winnipeg, Canada; 4 Kenya National Blood Transfusion Services, Nairobi, Kenya; 5 Centre for Virus Research, Kenya Medical Research Institute, Nairobi, Kenya; University of the Witwatersrand, SOUTH AFRICA

## Abstract

**Background:**

The rapid spread of HBV has resulted in the emergence of new variants. These viral genotypes and variants, in addition to carcinogenic risk, can be key predictors of therapy response and outcomes. As a result, a better knowledge of these emerging HBV traits will aid in the development of a treatment for HBV infection. However, many Sub-Saharan African nations, including Kenya, have insufficient molecular data on HBV strains circulating locally. This study conducted a population-genetics analysis to evaluate the genetic diversity of HBV among Kenyan blood donors. In addition, within the same cohort, the incidence and features of immune-associated escape mutations and stop-codons in Hepatitis B surface antigen (HBsAg) were determined.

**Methods:**

In September 2015 to October 2016, 194 serum samples were obtained from HBsAg-positive blood donors residing in eleven different Kenyan counties: Kisumu, Machakos, Uasin Gishu, Nairobi, Nakuru, Embu, Garissa, Kisii, Mombasa, Nyeri, and Turkana. For the HBV surface (S) gene, HBV DNA was isolated, amplified, and sequenced. The sequences obtained were utilized to investigate the genetic and haplotype diversity within the S genes.

**Results:**

Among the blood donors, 74.74% were male, and the overall mean age was 25.36 years. HBV genotype A1 (88.14%) was the most common, followed by genotype D (10.82%), genotype C (0.52%), and HBV genotype E (0.52%). The phylogenetic analysis revealed twelve major clades, with cluster III comprising solely of 68 blood donor isolates (68/194-35.05%). A high haplotype diversity (Hd = 0.94) and low nucleotide diversity (π = 0.02) were observed. Kisumu county had high number of haplotypes (22), but low haplotype (gene) diversity (Hd = 0.90). Generally, a total of 90 haplotypes with some consisting of more than one sequence were observed. The gene exhibited negative values for Tajima’s D (-2.04, p<0.05) and Fu’s Fs (-88.84). Several mutations were found in 139 isolates, either within or outside the Major Hydrophilic Area (MHR). There were 29 mutations found, with 37.9% of them situated inside the "a" determinant. The most common mutations in this research were T143M and K122R. Escape mutations linked to diagnostic failure, vaccination and immunoglobulin treatment evasion were also discovered. Also, one stop-codon, W163STP, inside the MHR, was found in one sample from genotype A.

**Conclusion:**

In Kenya, HBV/A1 is still the most common genotype. Despite limited genetic and nucleotide diversity, haplotype network analysis revealed haplotype variance among HBV genotypes from Kenyan blood donors. The virological properties of immune escape, which may be the source of viral replication endurance, were discovered in the viral strains studied and included immune-escape mutations and stop-codon. The discovery of HBsAg mutations in MHR in all isolates highlighted the need of monitoring MHR mutations in Kenya.

## Introduction

The Hepatitis B virus is the causal agent of HBV infection, which is spread by body fluid contact and causes both acute and chronic hepatitis infections. Chronic HBV infection can progress to hepatocellular carcinoma (HCC) and liver cirrhosis (LC), both of which are fatal [1, 2]. HBV has infected one-third of the world population, resulting in an estimated 296 million chronic infections and over 800,000 deaths in 2019, the majority of which were caused by LC and HCC [3]. Despite the availability of a vaccination for prevention, 1.5 million new infections are recorded each year [3].

Unlike other DNA viruses, HBV replication involves a critical reverse transcription step [4]. This stage requires ribonucleic acid (RNA)-dependent DNA polymerase, which lacks proofreading function, resulting in error-prone viral replication. Error-prone replication is a crucial molecular mechanism in the development of genotypes and sub-genotypes [5]. HBV is classified into ten genotypes, HBV-A to HBV-J, which differ by 7.5–15% at the nucleotide level of whole genomes [6]. Around 40 sub-genotypes with 4–7.5% divergence of the total genomic sequence are identified [7]. The outcome of HBV infections, responsiveness to therapy, and risk of HCC development have all been connected to viral heterogeneity [8].

For viruses to adapt to changing settings, genetic diversity is important [9]. Thus, DNA/RNA sequence analysis can be useful for understanding what factors shape genetic diversity patterns in natural populations [10, 11], which is important for monitoring the emergence of new variants [10, 12]. Since HBV reverse transcriptase lacks a proof-reading mechanism, a significant rate of mutation occurs spontaneously during viral genome replication [13, 14]. These alterations may have pathobiological consequences during immunosuppression-driven HBV reactivation, boosting viral replication re-uptake during the first weakening of immune responses [15–18].

There is little understanding of HBV types and their genetic diversity in Kenyan blood donors, with few publicly available surface gene sequences from Kenyan blood donors on the NCBI nucleotide database. Furthermore, the study investigated nucleotide heterogeneity and amino acid variations in the HBV surface genes between our samples (Kenyan sequences) and publicly available international sequences. Further it also determined the prevalence and characteristics of immune-associated escape mutations and stop codons in HBsAg.

## Materials and methods

### Study design and sampling

All donor blood units in this study were tested for HBV using the Murex HBsAg kit v.3 (Abbott Diagnostics, Louvain-la-Neuve, Belgium), a qualitative test intended for the detection of HBsAg, according to the National Blood Transfusion Service (NBTS) testing algorithm [19, 20]. A total of 194 de-identified serum samples, were obtained from NBTS headquarters in Nairobi after being screened positive by the national algorithm. Serology testing results, as well as demographic information, were recorded. All donors had verbally consented to their blood being used for research reasons. At the NBTS centers in the counties, staff collecting blood samples, sought verbal consent from donors, to use their sample for research purposes, in case they tested positive for HBV. Also, Individuals who tested positive for HBsAg were notified and referred for counselling and treatment. The 194 samples had been taken between September 2015 and October 2016 from eleven distinct counties: Eldoret (Uasin Gishu), Embu, Garissa, Kisii, Kisumu, Lodwar (Turkana), Machakos, Mombasa, Nairobi, Nakuru, and Nyeri. Serum samples were maintained at -80°C before being shipped to the National Microbiology Laboratory, Winnipeg, Canada, for further testing and characterization of HBV isolates. Molecular work was conducted between November 2016 and April 2017.

### Ethics approval

Ethical approval for the research was obtained from Kenya Medical Research Institute’s ethical review committee, approval number SERU 2209. Authors did not have access to information that could identify individual participants during or after data collection.

### DNA extraction, amplification and sequencing

Hepatitis B Virus DNA from HBsAg positive samples was extracted using the QIAamp® DNA blood mini kit (Qiagen Inc., Ontario, Canada). The extract, from 200μl serum, was eluted in 60μl nuclease free water (Ambion®, Thermo Fisher Scientific, Ontario, Canada). The partial HBV S gene was amplified as previously described by Stuyver et al., [21]. Briefly, 5μl DNA extract was amplified using primers (1st stage forward and reverse primers, 5ʹ-GGAGTCGTGCAGG TTTTGC-3ʹ, 5ʹ-TGCTGCTATGCCTCATCTTC-3ʹ; nested stage forward and reverse primers, 5ʹ-CARAGACAAAAGAAAATTGG-3ʹ, 5ʹ-CAAGGTATGTTGCCCGTTTGTCC-3ʹ); primers were prepared by IDT Canada, Ontario, Canada) specific for the HBsAg gene coding region (approx. nt 414–822, numbering based on GenBank access AY128092). Amplification proceeded using AmpliTaq Gold DNA Polymerase (Thermo Fisher Scientific) following the manufacturer’s suggested protocol and an annealing temperature of 45°C for each stage. Nested PCR products (340 bp) were gel purified and cycle sequenced using an Applied Biosystems 3730 XL DNA Analyzer (Thermo Fisher Scientific).

### Sequence alignment and phylogenetic analysis

Geneious Prime version 2022.2.2 software was used in *De novo* assembly of all the 194 sequences, and ClustalW algorithm was used for multiple sequence alignment. The sequence alignment was trimmed to 299 bp (approx. nt 455–753) for downstream analysis. The DNA Sequence Polymorphism (DnaSP) version 6.12.03 was used for genetic computations [22]. The software aided in determination of natural selection, genetic drift, mutation, recombination, and gene flow levels. Nucleotide diversity (π), haplotype diversity (Hd), pairwise population differences, Tajima’s D [23], and Fu and Li’s D*/F* [24] were computed to assess the null hypothesis that all mutations are selectively neutral [25]. The sequences were further mapped to accession number GQ183486 that was found to have most proximity to the Kenyan HBV sequences using ClustalW algorithm. CodonCode Aligner software (version 10.0.2) was used in revealing mutated amino acid codons. Further, amino-acid sequences were compared with known wild type (ALG02604.1), to analyze presence of mutations within the MHR. The mutation pattern was assessed based on published reports using GENETYX® ver.9 [26]. The median-joining haplotype network was constructed by PopArt version 1.7. The 194 isolate sequences from this study were deposited in NCBI (GenBank) and assigned the accession numbers ON832834–833075. Sequences were aligned with HBV genotype reference sequences (S1 Table) and trimmed using MAFFT v7 [27] and BioEdit v7.2.5 [28], respectively. Maximum likelihood analysis of the partial HBsAg-coding region (nested amplicon trimmed to 299 bp) was performed using IQ-Tree software [29] by the SYM+γ+I model determined as the most appropriate substitution model for the alignment. Phylogenetic branch support was computed by the approximate likelihood-ratio test based on a Shimodaira-Hasegawa-like procedure [30]. HBV genotype designation was based on clustering with reference sequences supported by branch support ≥ 75%. The tree was built with the neighbor-joining method (1000 replicate bootstrapping), and the branches were converted into a cladogram. The sequences were classified into twelve clades based on the results. Genotype A blood donor haplotypes, red circle; genotype C blood donor haplotype, green circle; genotype D blood donor haplotypes, blue circles; genotype E blood donor haplotype, yellow circle. Shared haplotype sequences are designated with a triangle in the color of their genotype.

## Results

### Characteristics of the study population

The demographic data, HBsAg-positive findings and collection dates were provided by NBTS (**Table 1**). The study comprised 194 blood samples from donors with a mean age of 25.36 years. Most samples were from males (145/194, 74.7%) as compared to females (49/194, 25.3%). The HBV case group were from 11 counties of Kenya, with 18.6% (36/194) from Kisumu county; 17.0% (33/194) collected from Machakos county; 16.5% (32/194) Eldoret (Uasin Gishu county); 13.9% (27/194) Nairobi; 7.7% (15/194) Nakuru; 6.7% (13/194) Embu; 5.7% (11/194) Garissa; 5.7% (11/194) Kisii; 5.2% (11/194) Mombasa; 1.5% (3/194) Nyeri; and 1.5% (3/194) Lodwar (Turkana county).

**Table 1:** Demographic data of blood donors.

Parameter		Sample size
Name	Level	
**Gender**	Males	74.7% (145/194)
	Females	25.3% (49/194)
**Age group**	16–25	126
	26–35	41
	36–35	17
	46–78	10
Mean age	25.36 (26–35)	

### Genetic diversity of the partial HBV large surface protein gene in Kenya

A total 91 segregating sites were observed in the S gene of 194 samples sequenced from Kenya. Kisumu County had 23.2% (45/194) of the 91 mutated sites, followed by Machakos at 19.1% (37/194), Eldoret at 16.5% (32/194), Nakuru at 15.5% (30/194), Embu at 14.9% (29/194), and Nairobi at 13.9% (27/194), with the rest falling below 13% (**Table 2**). The nucleotide diversity (π) per study site ranged from 0.004 to 0.04, with the highest being observed in S gene sequences of HBV isolates from Nyeri, which could have been influenced by the small number of sequences analyzed. The HBV S gene displayed high haplotype diversity (Hd) in Kenya (0.9), with variation ranging from 0.7 to 1 across the eleven study sites resulting in a total of 89 haplotypes (**Table 2**). Machakos had the highest Hd value (0.95), while Nakuru had the lowest (0.70). HBV isolates from Kisumu had the highest number of haplotypes (22), followed by Eldoret (20), Machakos (20), and Nairobi (14). The remaining Counties had fewer than ten haplotypes recorded (**Table 2**).

**Table 2:** Genetic diversity indices of surface protein gene from HBV isolate circulating in human population in 11 counties of Kenya.

Study site	n	Total mutations	Π	H	Hd	Tajima’s D	Fu’s Fs	Fu and Li’s D*	Fu and Li’s F*
Nakuru	15	30	0.02	8	0.70	-0.81	1.17	-0.40	-0.54
Nairobi	27	27	0.02	14	0.93	-0.88	-2.82	-0.05	-0.35
Embu	13	29	0.02	7	0.73	-2.10**	0.19	-2.65	-2.62
Eldoret	32	35	0.03	20	0.94	-0.39	-5.08	-0.40	-0.43
Machakos	33	37	0.01	20	0.95	-1.88	-10.38	-2.34	-2.40
Nyeri	3	20	0.04	3	-	-	-	-	-
Kisii	11	21	0.02	7	0.93	-1.34	-0.32	-1.65	-1.83
Garissa	11	22	0.03	9	0.96	0.89	-1.14	0.97	0.96
Mombasa	10	14	0.01	8	0.96	-1.60	-3.31	-1.79	-1.78
Lodwar	3	2	0.004	3	-	-	-	-	-
Kisumu	36	45	0.018	22	0.90	-1.77**	-9.163**	-2.08	-2.24
Kenya	194	91	0.02	90	0.94	-2.04*	-88.84***	-6.32**	-5.17**

n: sample proportion, π: Nucleotide diversity, h: Number of haplotypes, Hd: Haplotype diversity

### Population structure and gene flow of HBV in Kenya counties

Most of the genetic variation in the S gene of HBV from Kenya (88.1%) was observed within populations, with only 11.9% variation observed among virus populations from different Counties (**Table 3**).

**Table 3:** Molecular variance of the S gene in HBV circulating in Kenya.

Source of Variation	d.f	Sum of squares	Variance components	Percentage of variation (%)
Among populations	11	96.268	0.39763 Va	11.9
Within populations	183	535.898	2.94450 Vb	88.1
Total	194	632.166	3.34213	100

**Fig 1:**
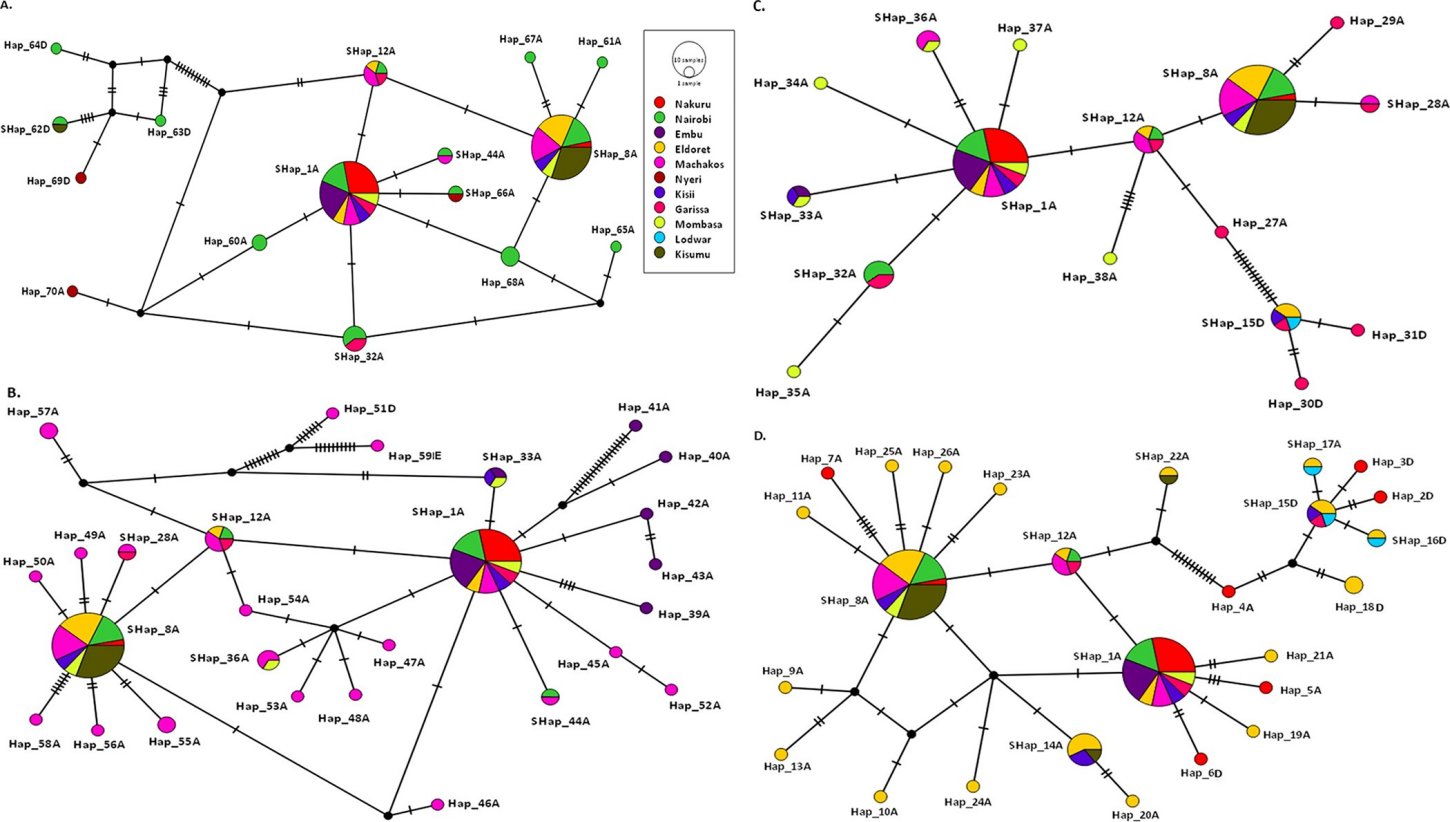
Local haplotype network of HBV based on S gene sequences from 11 counties in Kenya. The red circle represents sequences from Nakuru, Nairobi (green), Embu (violet), Eldoret (yellow), Machakos (purple), Nyeri (brown), Kisii (blue), Garissa (pink), Mombasa (orange), Lodwar (sky blue), and Kisumu (jungle green). A. haplotypes from Central (Nairobi and Nyeri), B. Eastern (Machakos and Embu), C. Northern and Coastal region (Garissa and Mombasa), D. Rift valley (Eldoret, Lodwar and Nakuru), and E. Western region (Kisumu and Kisii). The number of mutations resulting in a specific haplotype is represented by the hatch marks, and the size of the circle corresponds to the number of sequences or samples in a specific haplotype.

Despite the number of haplotypes observed varying by counties of patient origin, various mutated loci and haplotypes were shared across Counties. Samples showing identical sequences to those from other counties were coded as shared haplotypes (SHap). However, those which shared sequences within the same county, were referred to as haplotypes (Hap). Similarly, samples with distinct or unique sequences were also regarded as Hap. Shared haplotype SHap_8A of genotype A origin was found in each geolocation, accounting for 17% (33/194) of the sequences examined (**Fig 1**). Haplotype SHap_1A was a shared common ancestor, while haplotype SHap_8A was the most recent ancestor among Kenyan isolates (**Fig 1**). Other shared haplotypes among genotype A were SHap_1A at 16.5% (32/194), SHap_14A (3.6%, 7/194), SHap_12A (2.6%, 5/194), SHap_32A (2.6%, 5/194), SHap_33A (1.5%, 3/194), SHap_36A (1.5%, 3/194), SHap_22A (1%, 2/194), SHap_28A (1%, 2/194), and SHap_66A (1%, 2/194). Among genotype D, shared haplotypes included SHap_15D at 2.6% (5/194), SHap_16D (1%, 2/194), and SHap_62D (1%, 2/194). Shared genotype D haplotypes were only observed in Eldoret, Lodwar, Garissa, Nairobi, Kisumu, and Kisii (**Fig 1**). SHap_12A and Hap_74A served as a common ancestry link to genotype A, C, and D across Kenya’s eleven counties.

Kisumu and Machakos counties had the most shared mutated loci (29) followed by Eldoret and Kisumu (27) and Eldoret and Machakos (24) (**Table 4**). The computed low Fst value was observed between all study sites, with counties with higher Nm values recording the lowest Fst values (**Table 4**). Low Fst values indicate a lack of population structure and a lack of geographical or genetic barriers to gene flow. This increases heterogeneity within population but increases homogeneity (reduced diversity) among population. Nairobi and Kisii had the highest level of gene flow (Nm = 36.9), followed by Eldoret-Garissa (13.0), Nairobi-Eldoret (12.3), Eldoret-Kisii (12.0) and Nakuru-Nairobi (11.1) (**Table 4**).

**Table 4:** Hepatitis B virus population structure and gene flow in Kenya.

Population		No. of shared polymorphic sites	Fst	GammaSt	GammaSt Nm
Nakuru	Nairobi	18	-0.01	0.02	11.1
Nakuru	Embu	2	0.07	0.07	3.4
Nakuru	Eldoret	20	0.02	0.03	8.8
Nakuru	Machakos	18	0.06	0.05	4.3
Nakuru	Nyeri	17	-0.14	0.04	5.8
Nakuru	Kisii	18	-0.02	0.02	10
Nakuru	Garissa	18	-0.02	0.03	8.6
Nakuru	Mombasa	4	0.05	0.06	4.3
Nakuru	Lodwar	0	0.72	0.34	0.5
Nakuru	Kisumu	19	0.12	0.08	2.9
Nairobi	Embu	1	0.08	0.06	3.8
Nairobi	Eldoret	23	0.01	0.02	12.3
Nairobi	Machakos	22	0.01	0.02	10.0
Nairobi	Nyeri	19	-0.08	0.05	4.5
Nairobi	Kisii	19	-0.05	0.01	36.9
Nairobi	Garissa	21	0.02	0.04	5.8
Nairobi	Mombasa	5	0.02	0.03	8.5
Nairobi	Lodwar	0	0.80	0.34	0.5
Nairobi	Kisumu	25	0.08	0.05	4.4
Embu	Eldoret	3	0.17	0.09	2.5
Embu	Machakos	4	0.12	0.08	3.0
Embu	Nyeri	0	0.06	0.15	1.5
Embu	Kisii	2	0.05	0.07	3.5
Embu	Garissa	0	0.16	0.14	1.6
Embu	Mombasa	2	0.02	0.05	4.5
Embu	Lodwar	1	0.84	0.58	0.2
Embu	Kisumu	1	0.26	0.13	1.6
Eldoret	Machakos	24	0.05	0.04	5.9
Eldoret	Nyeri	19	-0.09	0.03	8.5
Eldoret	Kisii	19	0.003	0.02	12.0
Eldoret	Garissa	21	-0.02	0.02	13.0
Eldoret	Mombasa	7	0.11	0.05	4.7
Eldoret	Lodwar	2	0.70	0.20	1.0
Eldoret	Kisumu	27	0.03	0.03	8.0
Machakos	Nyeri	19	0.03	0.08	2.9
Machakos	Kisii	20	-0.01	0.02	10.3
Machakos	Garissa	21	0.09	0.08	3.0
Machakos	Mombasa	10	0.01	0.03	9.6
Machakos	Lodwar	0	0.84	0.38	0.4
Machakos	Kisumu	29	0.05	0.04	6.0
Nyeri	Kisii	18	-0.08	0.10	2.3
Nyeri	Garissa	18	-0.21	0.03	8.6
Nyeri	Mombasa	4	0.04	0.18	1.1
Nyeri	Lodwar	0	0.41	0.43	0.3
Nyeri	Kisumu	20	0.04	0.06	3.7
Kisii	Garissa	19	0.01	0.05	4.4
Kisii	Mombasa	7	-0.03	0.03	7.0
Kisii	Lodwar	0	0.81	0.55	0.2
Kisii	Kisumu	19	0.07	0.05	5.2
Garissa	Mombasa	5	0.12	0.11	2.0
Garissa	Lodwar	0	0.61	0.31	0.6
Garissa	Kisumu	22	0.09	0.07	3.5
Mombasa	Lodwar	0	0.88	0.70	0.1
Mombasa	Kisumu	8	0.17	0.07	3.2
Lodwar	Kisumu	0	0.80	0.28	0.6

Fst: Fixation index, Nm: Number of migrants

**Fig 2:**
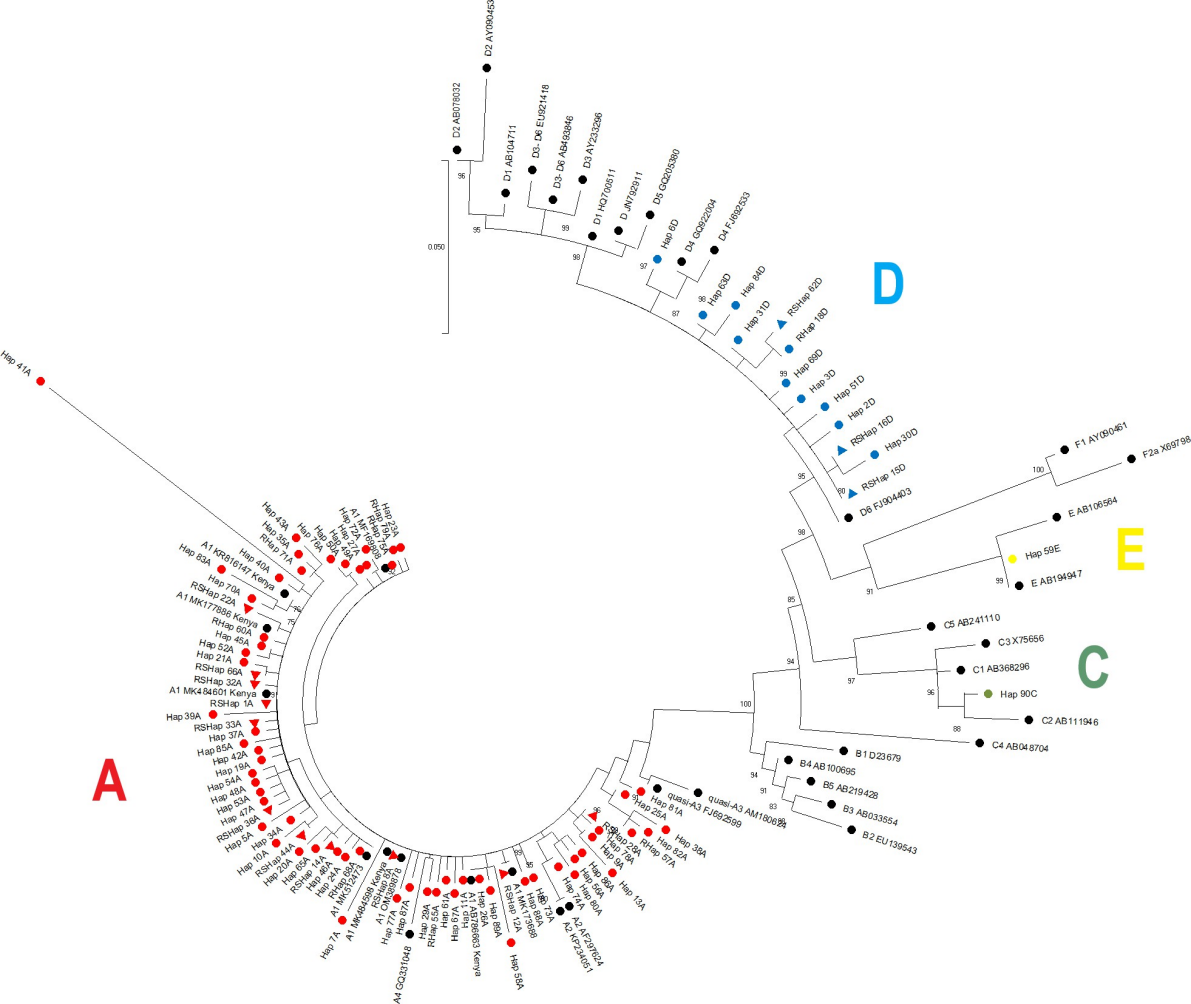
Phylogenetic analysis of HBV DNA positive blood donors. HBsAg subgenomic sequences (trimmed to 299 bp) were aligned with GenBank reference sequences representing HBV genotypes A to F, with the accession numbers provided (black circles); reference sequences from Kenya were noted. HBV genotype designations for blood donor haplotypes were based on clustering with genotype reference sequences. Genotype A blood donor haplotypes, red circle; genotype C blood donor haplotype, green circle; genotype D blood donor haplotypes, blue circles; genotype E blood donor haplotype, yellow circle. Shared haplotype sequences are designated with a triangle in the color of their genotype.

Phylogenetic analysis was performed on 87 selected blood donors, representing a total of 190 samples. Among them, 22 were representatives of Shap (RSHap) or Hap (RHap), with 65 distinct samples (Hap). Globally, the oldest shared common ancestry with Kenyan isolates was isolate with accession number A1_MF169808_from Ethiopia (Fig 2). Eight haplotypes: Hap 23A, Hap 79A, Hap 75A, Hap 72A, Hap 27A, Hap 49A, Hap 50A, and Hap 76A, diverged from a recent common a recent common ancestry with A1_MF169808_(Ethiopia), and were classified under clade 1. Clade 2 comprised of Hap 43A, Hap 35A, Hap 71A, Hap 41A and Hap 40A, and had A1_KR816147_(Kenya) as common ancestor. Clade 3 had Hap 83A, Hap 70A, Shap 22A, Hap 60A, Hap 45A, Hap 52A, Hap 21A, Shap 66A, Shap 32A, Shap 1A, Hap 39A, Shap 33A, Hap 37A, Hap 85A, Hap 42A, Hap 19A, Hap 54A, Hap 48A, Hap 53A, Hap 47A, Shap 36A, Hap 5A, Hap 34A, Hap 10A and Hap 44A, with a common ancestor being A1_MK177886_ (Eldoret, Kenya) and A1_MK484601_(Kenya). Clade 4 recent common ancestor was A1_MK512473_(Rwanda), and had Hap 20A, Hap 65A, Shap 14A, Hap 46A, Hap 24A and Hap 68A. Clade 5 involved Hap 7A, Shap 8A, Hap 77A and Hap 87A, which clustered with A1_MK484598_(Kenya) and A1_OM389878_ (South Africa). Clade 6 recent common ancestor was A4_GQ331048_(Belgium), A1_AB786663_ (Eldoret Kenya), A1_MK173688_Somali, and entailed Hap 29A, Hap 55A, Hap 61A, Hap 67A, Hap 11A, Hap 26A, Hap 89A, Hap 58A, Shap 12A, Hap 88A and Hap 73A. The remaining clades included clade 7 (Hap 74A, Hap 80A, Hap 56A and Hap 86A, clustering with A2_AF297624_ from South Africa and A2_KP234051_ from Belgium), clade 8 (A2_AF297624 from South Africa and A2_KP234051_ from Belgium grouped with Hap 13A, Hap 9A, Hap 78A and Shap 28A), clade 9 (Hap 57A, Hap 82A, Hap 38A, Hap 25A, Hap 81A and SHap_33A which clustered with quasi-A3_AM180624_ from Cameroon as well as with quasi-A3_FJ692599_ from Haiti), clade 10 (Hap 90C grouped with B1_D23679_(Japan), B2_EU139543_(China), B3_AB033554_(Indonesia), B4_AB100695_(Vietnam), B5_AB219428_(Philippines), C1_AB368296_(Japan), C2_AB111946_(Vietnam), C3_X75656_(Polynesia), C4_AB048704_(Australian Aborigine) and C5_AB241110_(Philippines), clade 11 (Hap 59C clustered with E_AB106564_(Ghana), E_AB194947_(Cameroon), F1_AY090461_(El_Salvador), and clade 12 (Shap 15D, Hap 30D, Shap 16D, Hap 2D, Hap 51D, Hap 3D, Hap 69D, Hap 18AD, Shap 62D, Hap 31D, Hap 84D, Hap 63D and Hap 6C, and were related with D6_FJ904403_(Tunisia), D4_GQ922004_(Canada) and D4_FJ692533_ collected from Haiti).

### Protein surface mutations

Alignment of the surface protein of the 194 isolated HBV alongside reference genotype A and E and subsequent sequence led to the detection of 65 amino acid exchanges. The percentage of samples with these mutations was 71.7% (139/194). In sum, 65 mutations were detected in 139 of the 194 samples analyzed. The overall S gene mutation rate of HBV among HBV DNA-positive sera was 115/169 (68.1%) within genotype A samples, 22/23 (95.7%) for genotype D, with genotype C reporting 3/3(100%) In this study, 19 of the 65 mutations (29.2%) were located within the “a” determinant, 73.7% (14/19) in the first loop (amino acid positions 120–137) and 26.3% (5/19) in the second loop (amino acid positions 139–147). The mutations in the ‘‘a” determinant region included; K122R, T123S, T125M, T126S, P127T, P127L, A128D, Q129P, Q129R, N131T, F134Y, F134L, F134V and P135H in the first loop of ‘‘a” determinant and the rest of substitutions including T140G, T140S, T143S, T143M, D144G occurred in the second loop of ‘‘a” determinant. Of the 19 mutations in the “a” determinant, T143M mutation was found in 9 different samples, all belonging to HBV genotype A. Outside the MHR, the study report presence of S193L mutations among 3 samples. Additionally, MHR substitution associated with immune escape, and found in current study were P127T, E164D, T125M, Q129P, and P127L. Six different samples had I110L mutations, 5 belonging to HBV/A genotype and 1 from HBV/C1 genotype. The K122R substitution was detected in 26 samples within the MHR. Furthermore, within the same region, dimorphic codon V168A was observed among 11 samples, as well as codon T114S among 13 samples. Apart from S gene mutation, 9 double mutations (9/262; 3.4%) were also found in the present study, and it involved HBV genotype A1 and C1. Double mutations were observed at amino acids positions A159D (Hap_41A; 1/1), K122R (5/26; Hap_25A, Hap_38A, Hap_57A, Hap_59C and Hap_82A), S113T (1/1; Hap_90C), T140G (1/1; Hap_41A) and V184A (1/6; Hap_59C), with unshared haplotypes only involved. Hap_41A experienced 2 double mutations (A159D and T140G), and Hap_59C had 2 mutations at positions V184A and K122R. Finally, 1 stop-codon, W163STP, within the MHR, was detected in one sample, belonging to genotype A.

## Discussion

This study used genetic diversity indices and haplotype analysis networking to analyze HBV genetic variation and distribution of corresponding haplotypes in blood donors from different counties in Kenya. Generally, based on S gene of the analyzed sequences, HBV isolates from Kenya blood donors exhibited low nucleotide diversity and high haplotype diversity. Exhibition of low nucleotide and high haplotype diversity hints at a possible trend in important functional genes in hepatitis viruses since similar findings have also been recorded on capsid protein VP3/VP1 genes in Hepatis A virus isolates from Palestine [31]. Similarly, this could also suggest that the HBV isolates with high homozygosity either possess a fitness advantage in the sampled regions within Kenya. Kisumu, Machakos, and Eldoret counties had the highest number of detected haplotypes compared to the other investigated counties, and the reasons for this could not be determined, therefore it remains a subject for further research. This study’s 71.7% mutation rate on HBV’s S region is greater than the previously reported rate of 42.3% among HBV isolates from donor blood [32]. Nonetheless, this was lower than another mutation analysis research from Kenya, which found that 100% (14/14) of the isolates had amino-acid alterations [33]. This disparity can be due to the large number of samples included in our investigation.

High variations on HBV S gene from Kenya blood donors were observed within isolates population as compared to between isolates population from different counties. The observed high degree of variation within isolates in a county and low heterozygosity between isolates from different counties is ascribed to low levels of population structure and high levels of gene flow across counties or a lack of barriers to gene flow or spread of HBV infections [34]. This is exacerbated by the movement of infected individuals from one county to another, whether for treatment or for other purposes like as commerce [35]. As a consequence, most HBV haplotypes based on S gene sequences in this research were shared or common among Kenyan counties.

Based on haplotype groupings, high number of HBV isolates among blood donors in Kenya were genotype A, subtype A1. Indeed, results from this study suggest that genotype A1 sequences from Africa are somewhat conserved within the HBsAg sub genomic region. The study’s findings on the high prevalence of HBV subtype A1 corroborates prior studies on subtypes of HBV that were isolated from donor blood within Nairobi County, liver disease patients, and drug users [36–40]. Within A1 subtype, 9 different clusters were observed, this suggest that there are minimal variations that might be of importance to the survival of the HBV within host in Kenya. The findings imply that the A1 HBV subtype has recently undergone population expansion or purifying selection processes that limit genetic diversity, such as a selective sweep or bottleneck, as previously seen in HAV [31], as evidenced by the negative Tajima D results.

Other HBV subtypes observed to be in circulation in donors’ blood in Kenya included C, D, and E. In contrast to the widely distributed A1 subtype, genotype C is strongly associated with development to advanced liver disease, compared to all other genotypes [41–44], was restricted to Kisumu, and occurred at a lower frequency as previously reported in Mombasa [45]. The subtype has been described to be prevalent (17.2%) among patients presenting with liver disease in Eritrea [46] and has also been isolated in Vietnam, Myanmar and Thailand and linked to serious liver disease [47, 48].

Genotype D was found in blood of donors from nine counties but not in Embu or Mombasa. Genotype D is known to have a better survival, immune evasion mechanisms and is associated with rapid progression of disease, and death related to liver disease than other genotypes [49–52]. Also, genotype D is associated with Occult hepatitis B infection (OBI) [53–55]. Based on influence of the observed mutations on antigen functionality, 19 mutations were traced to “a” determinant region of S antigen. The observed mutations in the “a” determinant region were more compared to mutations that were previously reported [32, 37, 56, 57]. Key mutation observed in “a” determinant region included non-synonymous change T143M, that was present in 9 different samples, all belonging to HBV genotype A which is also reported by other studies [32, 58]. Presence of T143M mutations in Kenya raises alarm since it is associated with antigenic property alteration, escape to vaccine, failed diagnostic assays [32, 40], and problem in HBIg therapy [59]. Other mutations in this region included K122R, T123S, T125M, T126S, P127T, P127L, A128D, Q129P, Q129R, N131T, F134Y, F134L, F134V and P135H in the first loop of ‘‘a” determinant and the rest of substitutions including T140G, T140S, T143S, T143M, D144G occurred in the second loop of ‘‘a” determinant. Other non-syn mutations that were observed in Kenya isolates include sE164G, sG119R, Q129R, and sS193L which have been associated with HBsAg diagnostic test escape, immune response escape, and HBIg escape [60–63].

The MHR substitutions associated with immune escape in this study included the K122R, P127T, E164D, D144G, T125M, Q129P, and P127l and corroborates isolates from other countries such as Iran and Turkey [40, 64–68]. In addition, the study identified a T114P mutation occurring within the MHR region but outside the “a” determinant region in three isolates. This mutation T114P occurs within MHR region but outside the “a” determinant region, and is known to affect the detection of the HBsAg and was observed at a frequency >3% more as compared to other studies [40]. Compared to previous reports by Moradi et al., [56], where I110L mutation was observed in only HBV/D genotype, this study reports presence of this mutation in six isolates from HBV/A (five isolates) and HBV/C (one isolate) genotypes. The mutation dimorphic codon I110L, is also liked to HBsAg diagnostic test escape mutations and immune response escape mutations [60–63].

Finally, 1 stop-codon, W163STP, within the MHR, was detected in one sample, belonging to genotype A. Stop-codons can determine the accumulation of truncated HBsAg in the endoplasmic-reticulum, thus inducing oxidative stress and in turn enhancing hepatocytes proliferation [69, 70]. The study findings contrast previous study where stop-codon were detected at 20 HBsAg-positions including 172 and 182 [71]. However, the modern HBV surface antigen assays are able to detect most MHR variants that failed to detect in the past failed detection [72], and in fact the substitutions we established in our study were among the cases identified through a positive HBsAg test result.

## Conclusion

Despite having considerable haplotypic variation, the S gene region of HBV isolates from Kenya had low nucleotide diversity. This study demonstrates for the first time the frequency and geographic distribution of HBV genotypes in Kenya among a large cohort of blood donors, with HBV genotypes A and D predominating. Furthermore, because of the high prevalence of HBV/A and HBV/D, progression to the chronic phase of the illness, including the formation of HCC, is a possibility for Kenyan HBV patients. The presence of HBsAg mutations in MHR in virtually all isolates highlighted the need of monitoring MHR mutations in Kenya. Moreover, escape mutations related with diagnostic failure, vaccination escape, and immunoglobulin treatment have been reported herein.

## Supporting information

S1 Table (XLSX)
